# Rituximab and Abatacept Are Effective in Differential Treatment of Interstitial Lymphocytic Lung Disease in Children With Primary Immunodeficiencies

**DOI:** 10.3389/fimmu.2021.704261

**Published:** 2021-09-09

**Authors:** Yulia Rodina, E. Deripapa, O. Shvets, A. Mukhina, A. Roppelt, D. Yuhacheva, A. Laberko, V. Burlakov, D. Abramov, G. Tereshchenko, G. Novichkova, Anna Shcherbina

**Affiliations:** ^1^Department of Immunology, Dmitry Rogachev National Medical Research Center of Pediatric Hematology, Oncology and Immunology, Moscow, Russia; ^2^Department of Pathology, Dmitry Rogachev National Medical Research Center of Pediatric Hematology, Oncology and Immunology, Moscow, Russia; ^3^Department of Radiology, Dmitry Rogachev National Medical Research Center of Pediatric Hematology, Oncology and Immunology, Moscow, Russia; ^4^Department of Hematology, Dmitry Rogachev National Medical Center of Pediatric Hematology, Oncology and Immunology, Moscow, Russia

**Keywords:** ILLD, primary immunodeficiency, children, treatment, rituximab, abatacept

## Abstract

**Background:**

Interstitial lymphocytic lung disease (ILLD), a recently recognized complication of primary immunodeficiencies (PID), is caused by immune dysregulation, abnormal bronchus-associated lymphoid tissue (BALT) hyperplasia, with subsequent progressive loss of pulmonary function. Various modes of standard immunosuppressive therapy for ILLD have been shown as only partially effective.

**Objectives:**

To retrospectively evaluate the safety and efficacy of abatacept or rituximab in treatment of ILLD in children with PID.

**Methods:**

29 children (median age 11 years) with various forms of PID received one of the two therapy regimens predominantly based on the lesions’ immunohistopathology: children with prevalent B-cell lung infiltration received rituximab (n = 16), and those with predominantly T-cell infiltration received abatacept (n = 17). Clinical and radiological symptoms were assessed using a severity scale developed for the study.

**Results:**

The targeted therapy with abatacept (A) or rituximab (R) enabled long-term control of clinical (A 3.4 ± 1.3 *vs.* 0.6 ± 0.1; R 2.8 ± 1 *vs.* 0.7 ± 0.05, p < 0.01) and radiological (A 18.4 ± 3.1 *vs.* 6.0 ± 2.0; R 30 ± 7.1 *vs.* 10 ± 1.7, p < 0.01) symptoms of ILLD in both groups and significantly improved patients’ quality of life, as measured by the total scale (TS) score of 57 ± 2.1 in treatment recipients *vs.* 31.2 ± 1.9 before therapy (p < 0.01).

**Conclusions:**

ILLD histopathology should be considered when selecting treatment. Abatacept and rituximab are effective and safe in differential treatment of ILLD in children.

## Highlights

1. ILLD histopathology must be considered when choosing a treatment.
2. Targeted immunosuppressive therapy is effective in achieving control of ILLD in patients with inborn errors of immunity

## Introduction

Primary immunodeficiencies (PID) comprise a heterogeneous group of more than 400 inherited conditions with associated immune dysfunctions (1). Although severe recurrent/chronic infections are the main cause of mortality and morbidity in PID, in recent years, research has focused on immune dysregulation manifesting with oncological and autoimmune or autoinflammatory conditions involving various organs and systems (2, 3).

Interstitial lymphocytic lung disease (ILLD) is a recently characterized nonmalignant PID complication (4). In a cohort of adult patients who had common variable immunodeficiency (CVID), the incidence of ILLD was reported as ~ 22% (5) and as high as 38–52% in monogenic immune dysregulation syndromes such as CTLA4 (6) and LRBA (7) deficiencies. Prophylactic antibacterial therapy and immunoglobulin (IG) replacement, although necessary to control infections, seem to have little effect on the risks of ILLD development (8, 9). Immune dysregulation in ILLD causes reactive bronchus-associated lymphoid tissue (BALT) hyperplasia (10), which manifests in several pathomorphological forms: follicular bronchiolitis (FB), nodular lymphoid hyperplasia (NLH), and lymphocytic interstitial pneumonia (LIP) (11). While asymptomatic at first, ILLD leads to progressive pulmonary fibrosis and eventual respiratory failure (12). Treatment of ILLD using various immunosuppressive drugs, including corticosteroids, azathioprine, cyclosporine A, cyclophosphamide, hydroxychloroquine, methotrexate, mycophenolate mofetil, and infliximab, leads to variable results ranging from partial/transient effects to no effects at all and has often been associated with adverse effects and increased rates of infection (13–17). Therefore, targeted ILLD therapy is needed. In small cohorts of adult PID patients, rituximab in combination with azathioprine has proven effective (18, 19). Recent studies have shown abatacept to be effective in controlling symptoms of LRBA and CTLA deficiency, including ILLD (20, 21). Yet, there have been few reports and no consensus on ILLD treatment, especially in children.

Herein, we present a retrospective analysis of the safety and efficacy of ILLD monotherapy with rituximab or abatacept, chosen based predominantly on the pathomorphological characteristics of lymphoid infiltration, as well as genetic defects, in a cohort of pediatric patients with PID.

## Materials and Methods

### Study Overview

We reviewed the charts of patients with PID and ILLD who had been treated at our institution between April 2014 and October 2020 and extracted demographic, immunologic, pathomorphological, and radiographic data. We also queried the patients’ charts for previous immunosuppressive therapy. In all cases, the diagnosis of PID had been made according to the ESID diagnostic criteria (22), and in most cases it had been confirmed genetically (**Table E1**, **Supplementary Material**).

The study’s inclusion criteria were histological and/or radiological diagnosis of ILLD, treatment with rituximab or abatacept for at least 12 months, no signs of ongoing infectious pulmonary process at the beginning of treatment, and regular IG substitution and trough IgG levels > 5g/l. The exclusion criteria were lack of adherence to the therapy and the use of additional immunosuppression during the period analyzed.

Included in the study were the charts of 29 patients (9 boys and 20 girls) who had the following PID diagnoses: ataxia telangiectasia (AT) with the *ATM* gene defect (8/29), CTLA4 deficiency (*CTLA4*) (4/29), combined immunodeficiency (CID) without genetic verification (8/29), del22 syndrome (3/29), NFkB1/2 defect (*NFkB1/2*) (2/29), LRBA deficiency (*LRBA*) (2/29), Nijmegen syndrome (*NBS*) (1/29), Artemis deficiency (*DCLRE1C*) (1/29) (**Table E1**, **Supplementary Material**).

The median age at the beginning of treatment was 11.0 (3–17) years. The median age at the first clinical signs of ILLD was 7 (2.5–14) years.

The efficacy of the ILLD treatment was assessed after 12 months of treatment. In patients who continued the treatment for more than 14 months, a long-term follow-up assessment was performed at the time of data censoring (October 2020) or at the time of hematopoietic stem cell transplantation (HSCT).

### Preceding Therapy

According to the inclusion criteria, during the period analyzed, all patients had received regular IG replacement (0.4–0.6 g/kg every 4 weeks) with a mean trough IgG level of 7.67 ± 0.66 g/l (5.15–20 g/l).

Of the 29 patients, 17 had previously received immunosuppressive therapy for ILLD or other immune complications (**Table E1**, **Supplementary Material**) using azathioprine (3), mycophenolate mofetil (5), sirolimus (3) or adalimumab (1), methotrexate (2), and rituximab (2), either with no or only partial resolution of the ILLD symptoms. In addition, 14 patients also received corticosteroids (1–2 mg/kg/day) for 1–3 months and experienced the resolution of some lesions. The corticosteroid therapy had been accompanied by the following side effects: Cushing syndrome (14/14), osteopenia (10/14), and systemic bone loss (2/14). After cessation of corticosteroid therapy, all 14 patients had experienced ILLD relapse/progression.

### ILLD Severity Evaluation Scale

The severity of the main clinical symptoms (cough, rails, dyspnea) was evaluated using a severity scale we designed (**Table E2**, Online Repository), on which the assessment scores of the symptoms ranged from 0.5 (complete lack of symptoms) to 6 (maximal intensity). The severity of the disease was scored before the initiation of rituximab/abatacept therapy and again after 3, 6, and 12 months of treatment.

The initial radiological ILLD symptoms and their dynamics during treatment were evaluated using multi-slice computed tomography (CT). Because there are no standard criteria for radiological evaluation of ILLD symptoms (23, 24), we developed an algorithm for use in this CT-based evaluation (**Table E3**, **Supplementary Material**). The following changes were scored: focal lesions, presence of patches of diffuse ground-glass opacity indicative of interstitial changes, linear and reticular changes of interlobar interstitium or fibrosis, lobe pneumatization, and hilar/mediastinal lymph node size. Each sign was assigned a point score ranging from 0 to 3, each lung lobe was assessed separately, and the maximum possible number of points was 49.

The CT changes characteristic of ILLD varied among patients and included three major types of lesions: ground-glass opacities or cotton-like foci, which are typical of FB; large sarcoid granuloma-like foci, typical of NLH; and diffuse interstitial changes, typical of LIP (**Figure E1**, **Supplementary Material**).

Due to the age restriction and the disease clinical manifestations in some patients (i.e., myopathy and polyneuropathy in AT patients), spirometry was not performed in the majority of patients; therefore, its results were not included in the analysis.

The parameters of the blood gases were evaluated using the standard method and an ABL800 Flex blood gas analyzer (Radiometer, Denmark).

### Morphology and Immunohistochemistry and Therapy Tactics

The lung tissue pathomorphology was evaluated using standard H&E staining, and immunohistochemistry was evaluated using a set of markers that included CD20, Pax5, CD3, CD4, CD8, TdT, and Ki67. In all cases, *in situ* hybridization for the Epstein-Barr virus (EBV) marker EBER ISH Blue was performed.

To verify the ILLD diagnosis and define its form, a thoracoscopic lung biopsy was performed for most (22/29) patients. In the seven patients in whom biopsy was not possible due to the high risks associated with the procedure and/or the refusal of the parents, the ILLD form had been assigned based only on the radiological changes. The following pathomorphological forms of ILLD were identified: NLH (3/29), LIP (8/29), mixed changes characteristic of both FB and NLH (12/29), and FB and LIP (6/29) (**Table E1**, **Supplementary Material**).

According to the immunohistology, in all cases of abnormal BALT follicular hyperplasia (seen in both FB and NLH), predominantly B-cell (CD20+) proliferation was noted, while the diffuse interstitial changes seen in LIP were associated predominantly with T-cell (CD3+) infiltration.

Based on the predominant type of lymphocytic infiltration of the lungs (B or T lymphocytes), patients received either anti-CD20 therapy directed at mature B-cells (rituximab) or the T-cell costimulatory molecule inhibitor abatacept. For the analysis, patients were divided into two groups according to the treatment received.

Group 1 patients received rituximab at 375 mg/m^2^ as 4 weekly, consecutive intravenous (IV) infusions, with subsequent infusions of 375 mg/m^2^ performed every 3 months for 12 months.

Group 2 patients received abatacept at 10 mg/kg as 2 biweekly IV infusions, and then every 4 weeks for 12 months.

Four patients (P2, P4, P7, and P11) originally treated using rituximab (Group 1) reached only partial remission. Later, they were treated using abatacept. These patients were analyzed in both Groups 1 and 2, and their ILLD symptoms were assessed at the beginning of each respective treatment.

### Quality of Life Evaluation

The patients’ quality of life was evaluated using an adapted Russian-language version of the Pediatric Quality of Life Inventory (PedsQL) 4.0 questionnaire both before the rituximab/abatacept treatment and after 12 months of therapy. Physical functioning (PF), psychosocial functioning (PSF), and total scale scores (TS) were evaluated.

### Statistical Analysis

Statistical analysis of the data was performed using the XLSTAT Addinsoft 2017 software. The changes in the clinical and radiological signs were analyzed using the Mann–Whitney U test. The difference was considered statistically significant if р < 0.05.

### Ethics Statement

This retrospective study (ClinicalTrials.gov ID: NCT04572620) was approved by the Ethics Committee of the Dmitry Rogachev National Medical Research Center of Pediatric Hematology, Oncology, and Immunology.

## Results

**Figure 1:**
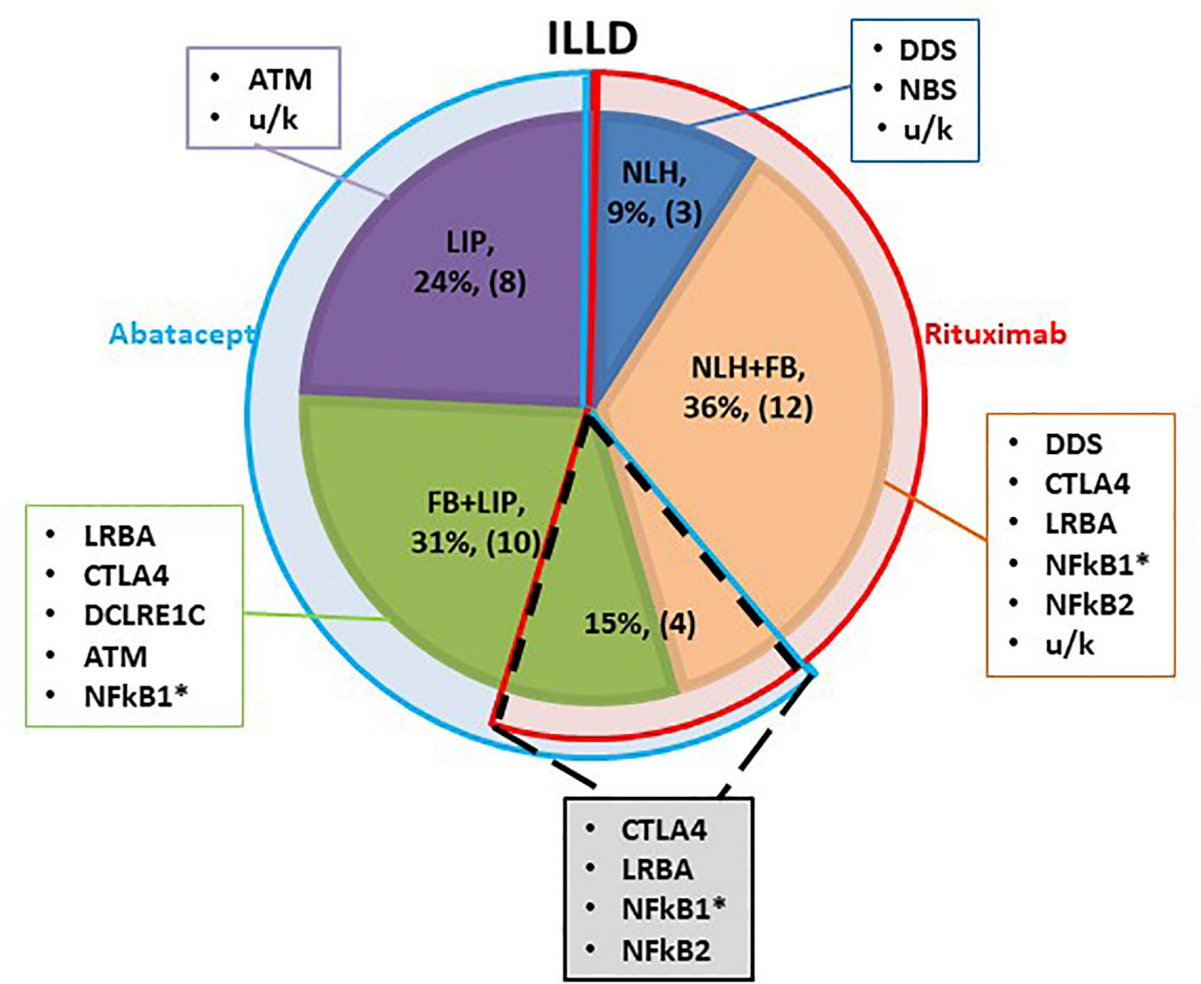
Schematic characterization of the ILLD patient groups. Shaded segments represent different histological types of ILLD, with genetic PID defects noted in corresponding rectangles. Blue semi-ring denotes patients treated with abatacept, red - with rituximab. Overlapping segment (dotted line) denotes patients who were treated with rituximab, and later with abatacept. * - biopsy was performed twice.

Most patients were still in the process of genetic PID verification at the time of the ILLD diagnosis, therefore the choice of targeted preparation was primarily based on the histological and radiological findings. It was hypothesized that ILLD with predominant B lymphocyte infiltration will respond to rituximab. Yet, two patients with predominant T cell infiltration and LIP histology were previously treated with rituximab for other autoimmune complications and no ILLD improvement was noted. Hence it was hypothesized that abatacept might be a preparation of choice in the LIP group. Three patients with NLH, 12 with NLH+FB and one with FB+LIP received treatment with rituximab, 8 patients with LIP and 10 with FB+LIP received abatacept, and 4 patients with NLH+FB/FB+LIP received consecutive treatment with rituximab and abatacept (**Figure 1**).

### Pathomorphological and CT Characteristics of ILLD

Group 1 (Rituximab) consisted of 11 girls and 5 boys whose median age was 12 (6–18) years, and the PID/genetic defect distribution was as follows: del22 (3/16), NFkB1/2 (*NFkB1/2*) deficiency (2/16), CTLA4 (*CTLA4*) deficiency (1/16), Nijmegen syndrome (*NBS*) (1/16), LRBA (*LRBA*) deficiency (1/16), Artemis deficiency (*DCLRE1C*) (1/16), and CID without genetic defect (7/16).

The morphological and/or radiological changes in this group were characterized as a combination of FB and NLH (12/16), NLH (3/16), and a combination of FB and LIP (1/16) (**Table E1**, **Figure E1**, **Supplementary Material**).

Group 2 (Abatacept) included 12 girls and 5 boys whose median age was 10 (5–17) years, and the PID/genetic defects distribution was as follows: AT (*ATM*) (8/17), CTLA4 deficiency (*CTLA4*) (4/17), LRBA deficiency (*LRBA*) (2/17), NFkB1/2 deficiency (*NFkB1/2*) (2/17), and CID without genetic defect (1/17).

The morphological and/or radiological changes in Group 2 were characterized as LIP (8/17) or a combination of LIP and FB (9/17) (**Table E1**, **Figure E1**, **Supplementary Material**).

**Table 1:** The ILLD variants and response to therapy in Groups 1 and 2.

Variant of ILLD	Patients, n	Complete remission, n	Partial remission, n	No effect, n	On preparation*, n
Group I					
NLH	3	3	0	0	0
FB+NHL	12	6	6	0	2
FB+LIP	1	0	0	1	0
Group II					
LIP	8	8	0	0	4
FB+LIP	9	9	0	0	6

FB, follicular bronсhiolitis; LIP, lymphoid interstitial pneumonia; NLH, nodular lymphoid hyperplasia. *Follow up 10 ± 2 mo, n, number of patients.

### Dynamics of the Clinical and Radiological Symptoms of ILLD in Patients Treated With Rituximab (Group 1)

**Figure 2:**
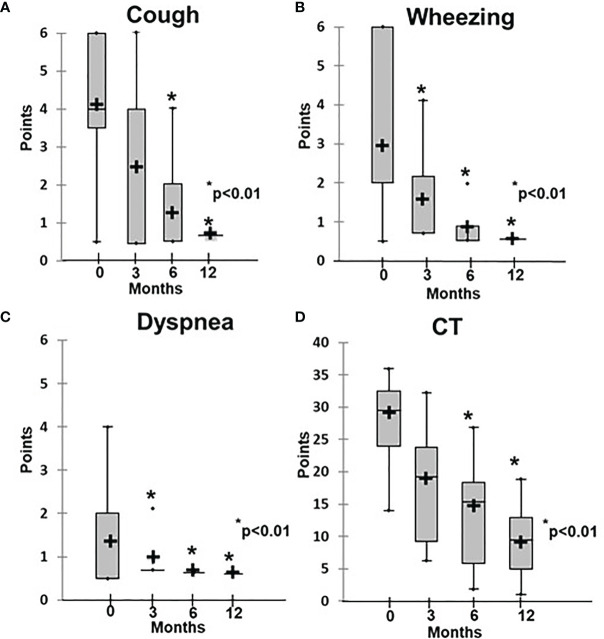
Dynamics of clinical and radiological ILLD symptoms in patients treated with rituximab. **(A)** Cough, **(B)** wheezing, **(C)** dyspnea, **(D)** CT changes.

**Figure 3:**
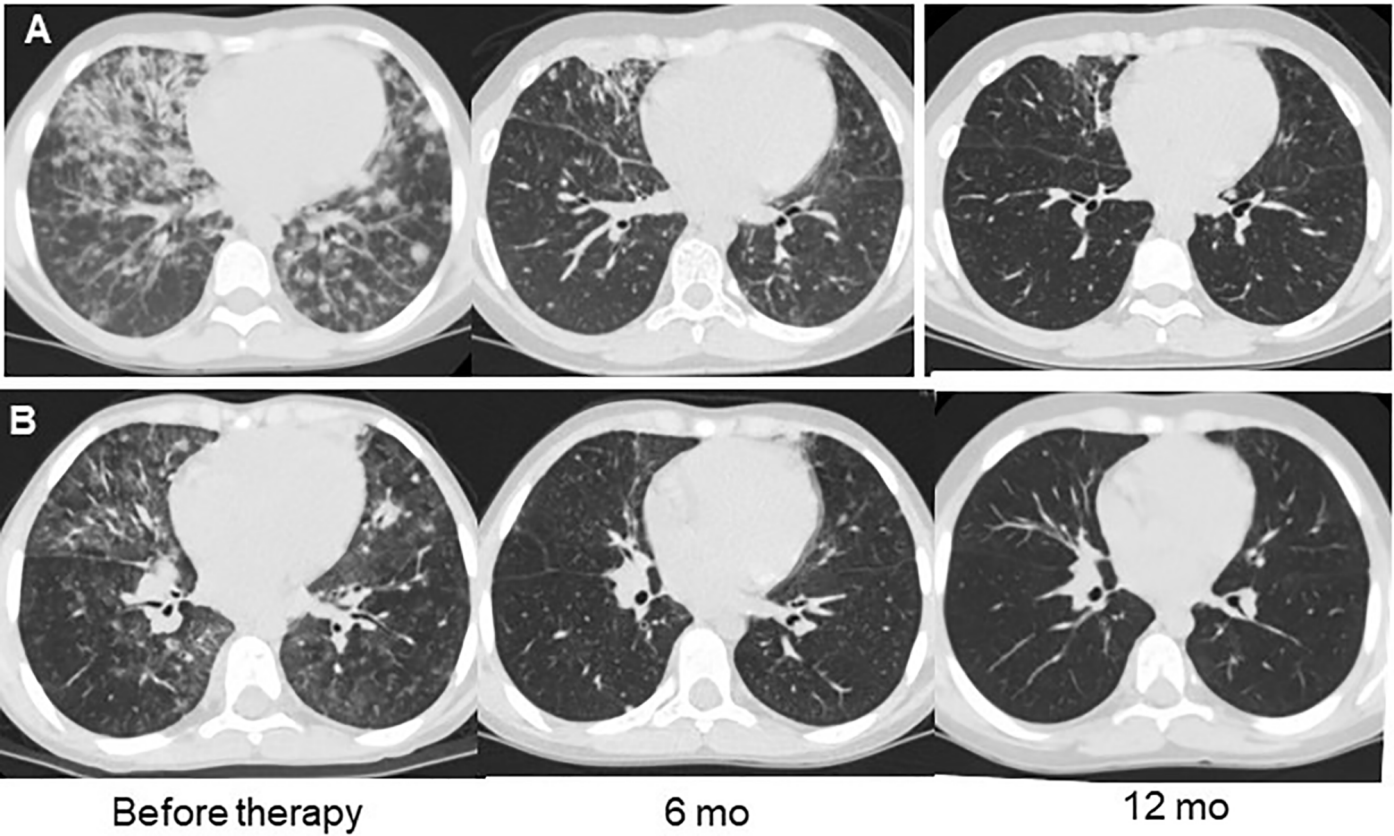
Examples of lung CT dynamics in the course of rituximab therapy. **(A)** – P8 with DiGeorge syndrome (del22); **(B)** – P14 with combined immunodeficiency with unknown genetic defect.

Before initiation of rituximab, the symptoms of the patients in Group 1 varied in intensity from subclinical (3/16) to chronic respiratory failure (3/16), including Grade 1 in two patients and Grade 3 in one patient. At the baseline, the average disease severity was 2.8 ± 1.0, which improved significantly to 1.3 ± 0.5 after 4 weeks of rituximab therapy (p = 0.03) and to 0.7 ± 0.05 at 6 months (р < 0.01) (**Figure 2**). The average radiological score at baseline was 30 ± 7.1, which during treatment slowly improved to reach significance at 6 months, while severity decreased to 10 ± 1.7 by 12 months (p < 0.01) (**Figures 2**, **3**).

Before therapy, abnormal blood gases and oxygen saturation (SaO_2_) were observed in three patients who had respiratory failure. Before treatment, a minimal SaO_2_ value of 75% (average SaO_2_ 95 ± 11%; normal range 98–100%) and рСО_2_ value of 55 mm Hg (average 51 ± 3.5 mm Hg; normal range 35–45 mm Hg) were observed in a patient who had Grade 3 respiratory failure, and the other two patients who had Grade 1 respiratory failure had SaO2 values of 95–96% and pCO2 values of 43–48 mm Hg. After 12 months of treatment, these values returned to normal ranges.

After 12 months of treatment, full remission was reported in 9 patients, partial remission in 6, and no effect in 1 patient. Of the six patients who achieved partial remission, two (P15, P16) had minimal CT findings of FB at 12 months and continued therapy with rituximab (**Table 1**). In the remaining four patients (P2, P4, P7, and P11) who originally had NLH+FB ILLD, all the NLH and most FB abnormalities resolved; however, radiological changes in the form of interstitial damage became obvious, and the patients were then classified as FB+LIP (**Figure E2**, **Supplementary Material**). These patients were switched to abatacept and achieved complete remissions (described in the next section). In one patient (P6) who had FB+LIP, rituximab had no effect on the ILLD symptoms. Due to the severity of the disease, this patient subsequently underwent HSCT and died from post-transplant complications.

### Dynamics of the Clinical and Radiological Symptoms in ILLD Patients Treated With Abatacept (Group 2)

In contrast to Group 1, most patients in Group 2 (16/17) had evident respiratory symptoms, with an average clinical score of 3.4 ± 1.3. Grade 1 respiratory failure was present in three cases, and Grade 2 was present in one case. Only one patient had asymptomatic CT changes.

**Figure 4:**
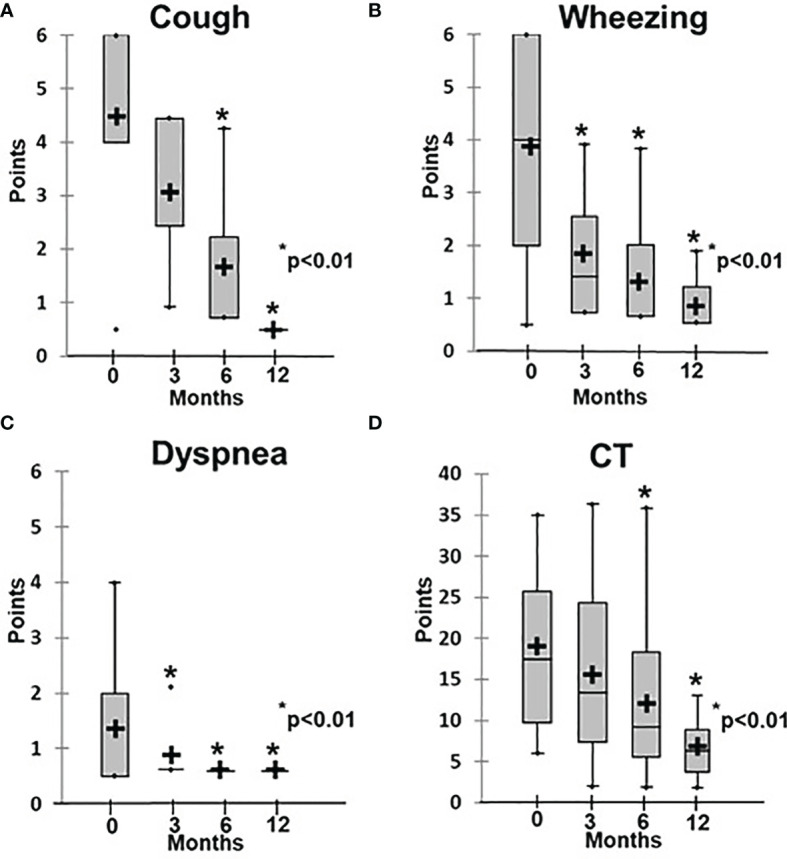
Dynamics of clinical and radiological symptoms in patients treated with abatacept. **(A)** Cough, **(B)** wheezing, **(C)** dyspnea, **(D)** CT changes.

**Figure 5:**
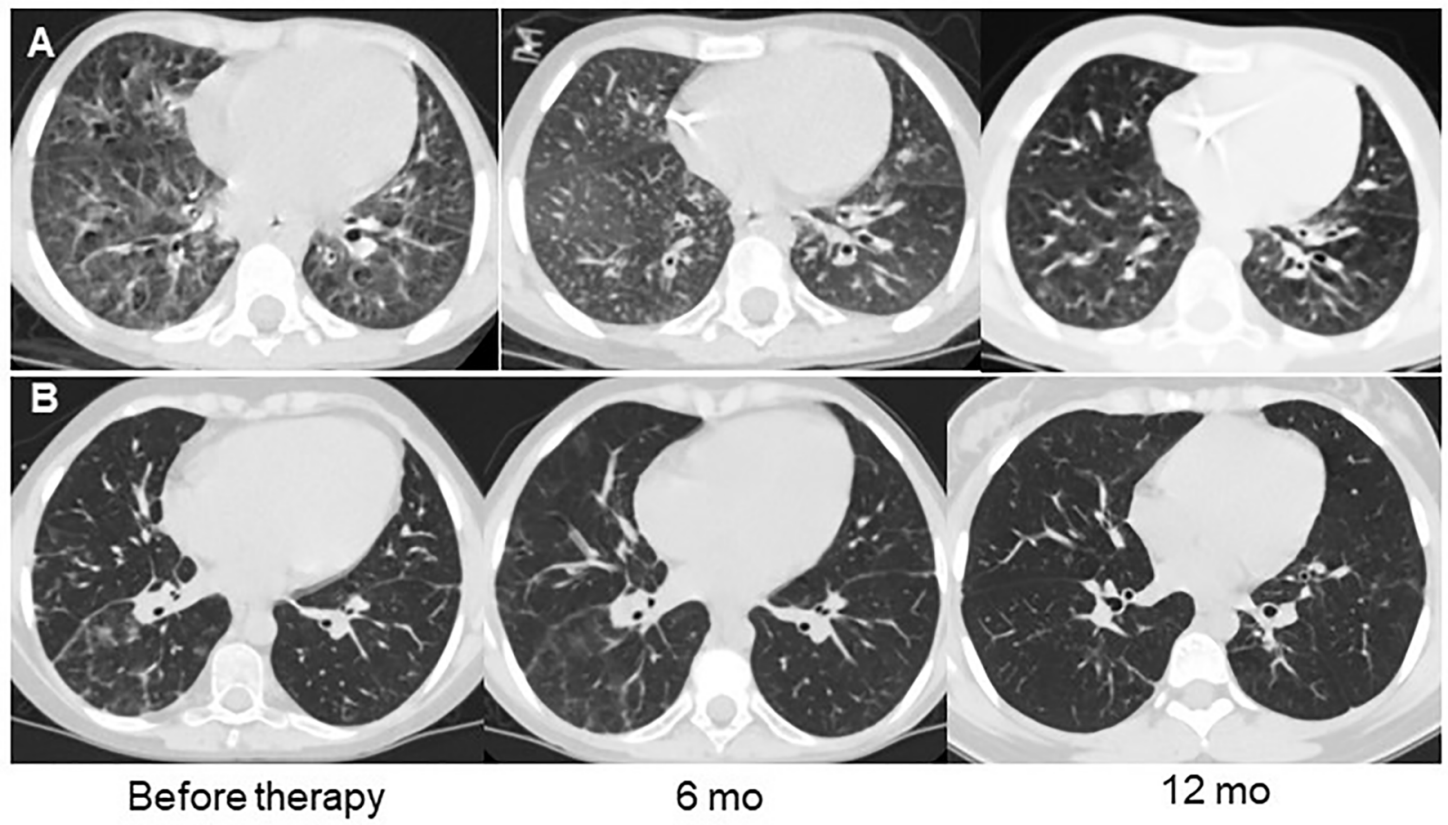
Examples of lung CT dynamics in the course of abatacept therapy. **(A)** – P28 with CTLA4 deficiency, **(B)** – P27 with ataxia-telangiectasia.

Patients in Group 2 demonstrated symptom changes that were much slower than in Group 1, especially regarding the radiological signs of ILLD. While their clinical symptoms, including cough, respiratory failure, and rails, improved after six months of abatacept therapy and the average score decreased from 1.2 ± 0.3 to 0.6 ± 0.1 (p < 0.01), their CT changes were even slower: Significant increases in lung pneumatization and the disappearance of or substantial decreases in the ground-glass symptoms, fibrosis, and peribronchial changes were observed only after 12 months of treatment, and the average score decreased from 18.4 ± 3.1 to 6.0 ± 2.0 (p < 0.01) (**Figures 4**, **5**).

After 12 months of treatment, full remission was reported in all 17 patients (**Table 1**).

In four patients, abnormal SaO_2_ (average 93.5 ± 0.9%) and рСО_2_ values (average 52 ± 4.7 mm Hg) and signs of respiratory failure were noted. These values normalized after six months of treatment.

Two patients from Group 2 (P17, P23) had previously received rituximab (four weekly doses) for other complications, including Coombs hemolytic anemia, lymphoproliferative syndrome, and persistent EBV-viremia. No improvements in their ILLD were noted after their short courses of treatment.

### Adverse Events

No cases of therapy intolerance or serious adverse effects of rituximab or abatacept were recorded during treatment. During the first two rituximab infusions, four patients had fevers that completely resolved after treatment with nonsteroid anti-inflammatory drugs. These events constituted a rate of 6.7% of all rituximab infusions.

### Quality of Life

**Figure 6:**
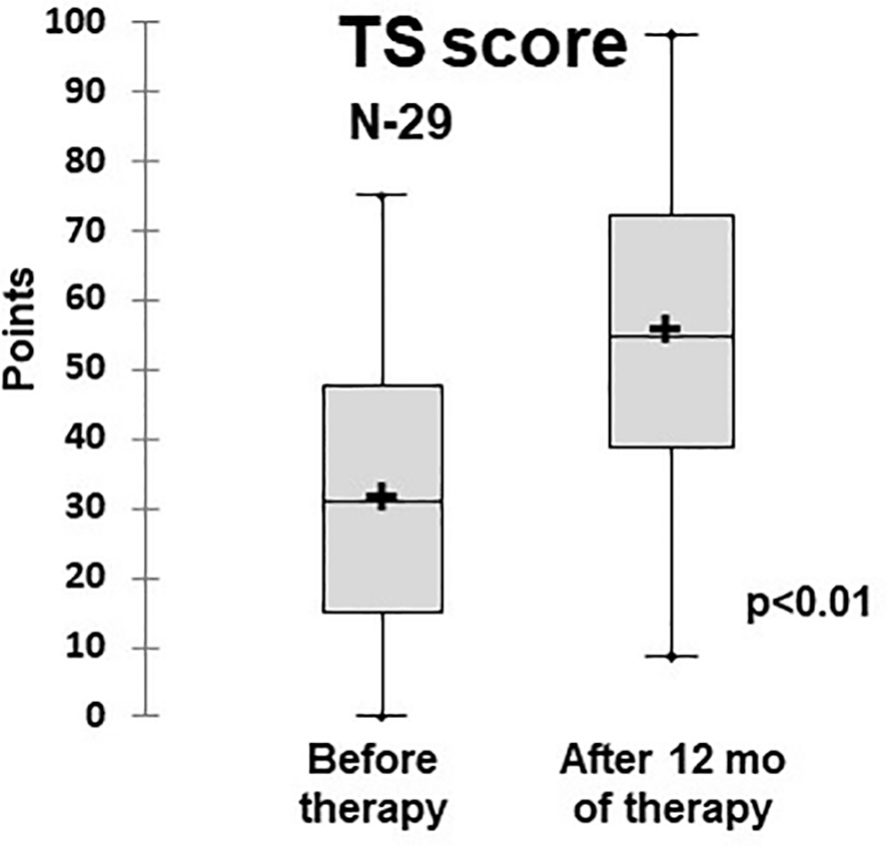
Dynamics of the quality of life (total scale score) in ILLD patients treated with abatacept or rituximab (PedsQL 4,0). N- number of patients.

The quality of life assessment demonstrated that patients who had PID and ILLD had physical and psychosocial function impairments: Before the treatment, the average TS score was 31.2 ± 1.9 points. The treatment provided statistically significant improvements to the physical and psychosocial aspects, leading to increases in specific and overall scores, with the TS score at 12 months being 57 ± 2.1 (**Table 2** and **Figure 6**).

**Table 2:** Results of the quality of life evaluation using PedsQL 4,0.

	PF score	PSF score			TS score
		EF	SF	CF	
Prior to treatment	31.1 ± 2.7	51.5 ± 3.6	30.4 ± 3.6	16.8 ± 2.4	31.2 ± 1.9
After 12 months of treatment	51.2 ± 3.7	80.3 ± 2.7	52.4 ± 3.8	45.8 ± 3.0	57 ± 2.1
P	p<0.001	p<0.001	p<0.001	p<0.001	p<0.001

TS, total scale score; PF, physical functioning; PSF, psychosocial functioning; EF, emotional functioning; SF, social functioning; CF, cognitive functioning, P- the score point difference between initial value and the one obtained at the screening point is statistically significant (p<0.01).

### Long-Term Follow-Up

At the data censoring point, 21/29 patients were alive, 2 of them had undergone successful allogeneic HSCT and were free of symptoms, including ILLD symptoms, and 5 were lost to follow up after 12 or more months of therapy. Three patients died in the early post-transplant period due to non-ILLD-related complications after having received HSCT. Therefore, 8 patients in Group 1 and 11 patients in Group 2 were available for long-term follow-up analysis (> 12 months), with the median follow-up time being 43.5 months (14–56 months). From Group 1, 6/8 patients were off rituximab therapy and in complete ILLD remission, and 2/8 were still receiving treatment (**Table E1**, **Supplementary material**). From Group 2, 1/11 was off therapy with no signs of ILLD, and 10/11 were still receiving treatment. In three of these ten, attempts to discontinue abatacept treatment had resulted in the return of ILLD symptoms, and therapy had been re-established.

## Discussion

Recent years have been marked by the recognition of the interstitial lymphocytic lung disease as a severe complication of many PIDs. As reported in a consensus statement from specialists in internal diseases, pulmonology, and clinical immunology in the UK (25), ILLD is severely underdiagnosed in adult PID patients due to the lack of widely accepted protocols for its diagnosis and management. To our knowledge, this is the first published observation of ILLD in a large cohort of pediatric patients, so its prevalence in pediatric PID is even more difficult to assess. As in adult PID patients (14), the effect of “classic” immunosuppressive therapies (local and systemic steroids, azathioprine, methotrexate, sirolimus, mycophenolate mofetil, and adalimumab) in our ILLD cohort had been unsatisfactory. Therefore, new treatment modalities are required.

Immune dysregulation leading to impaired mucosal tolerance is considered the leading mechanism of ILLD development (11, 26). However, the radiological and histomorphological pictures of ILLD vary among patients, making it difficult to classify for diagnostic and treatment purposes (27). As suggested previously, the cellular composition of the hyperplastic BALT and interstitial infiltrates might provide the basis for such a classification, as well as for the stratification of targeted ILLD treatments (28).

The patients analyzed in the current study were differentially treated with abatacept or rituximab, based predominantly on the histologically proven or radiologically predicted prevalence of T- or B-lymphocytes, respectively, in their lung infiltrates.

We demonstrated the dramatic effects of both treatments in their respective groups. In rituximab recipients, the response was observed after the very first month of treatment, while in abatacept recipients, significant clinical and radiological improvements were observed only after six months of therapy or even more. In our opinion, this may be due to the more extensive lung damage in LIP as compared to the focal lung lesions found in FB and NLH patients. In addition, the rituximab dose used in our patients was derived from the B-cell lymphoma treatment protocols aimed at the rapid elimination of tumor B-cells (29, 30), whereas the abatacept dose was initially developed to treat rheumatoid arthritis (31) and might not have been high enough to rapidly control the immune-mediated damage in patients with inherited immune dysregulation syndromes. We suggest that further studies aim to optimize the abatacept doses and regimens used to treat immune dysregulation in general and ILLD in particular.

We also suggest that in addition to histopathology, the response to ILLD therapy is determined by the underlying genetic defect. In our cohort, the most aggressive ILLD course was seen in patients who had profound T-cell dysregulation defects (*CTLA, NFkb1/2*, and *LRBA* deficiencies) and in AT patients, which is in accordance with the results of previous studies (7, 21, 22, 32). Four patients who had immune dysregulation PID were originally treated and responded quickly, but only partially to rituximab. All were subsequently treated with abatacept and achieved full ILLD remission. More studies are needed to investigate if abatacept can be used as a monotherapy in cases of ILLD with histological findings of NLH or NLH+FB and underlying defects of immune dysregulation (for instance, CTLA or LRBA deficiency), or a course of rituximab is initially required to quickly reduce the pathology caused by B cell hyperproliferation in these cases.

Due to the retrospective character the study had certain limitations. For instance, most patients did not receive treatment with both abatacept and rituximab to prove superiority of one treatment over the other based on the pathomorphological approach. Yet, two patients with LIP histopathology had previously received rituximab, and no improvement in the course of ILLD was noted, whereas abatacept treatment was subsequently successful. In our opinion it is highly suggestive that LIP changes do not respond to the B cell targeted treatment.

There is also a question regarding the optimal duration of treatment and/or long-term therapeutic modification. In our study, while rituximab was discontinued in most patients successfully, without any ILLD relapse, most patients in whom abatacept treatment was discontinued demonstrated ILLD progression and required continuous treatment. These were mostly patients who had AT, for which no curative treatment is currently available. Hence, the efficacy and safety of long-term—potentially lifelong—abatacept treatment require further investigation.

In the current study, treatment with either abatacept or rituximab was accompanied by only a few adverse effects, which were reversible. This suggests that these regimens are not only clinically effective but also safe, in stark contrast to the preceding steroid treatment, which had been accompanied by significant side effects.

Many patients in our group had predominantly radiological symptoms at the time of their ILLD diagnoses. Yet, given that other patients, some as young as 2.5 years old, already had signs of respiratory failure due to ILLD, awareness of immune lung diseases in children who have PID, early diagnosis, and early treatment are required to prevent severe lung damage and to improve the quality of life, which we determined to be significantly affected by ILLD.

In conclusion, the use of abatacept and rituximab as targeted monotherapies demonstrated sufficient efficacy and safety in the treatment of children who had ILLD-complicated PID. Questions of the optimal therapy duration and/or modification of the treatment regimen, which have been raised by this and previous studies, should be resolved based on the results of longer-term follow-up and subsequent prospective studies.

## Data Availability Statement

The original contributions presented in the study are included in the article/**Supplementary Material**. Further inquiries can be directed to the corresponding author.

## Ethics Statement

The studies involving human participants were reviewed and approved by The Ethics Committee of the Dmitry Rogachev National Medical Research Center of Pediatric Hematology, Oncology, and Immunology. Written informed consent to participate in this study was provided by the participants’ legal guardian/next of kin.

## Author Contributions

All authors contributed to the article and approved the submitted version. AS drafted the manuscript, and contributed to concept and design, critical revision of the intellectual content, and final approval. GN contributed to concept and design, critical revision of the intellectual content, and final approval YR wrote the manuscript, created the tables and figures. DA performed pathomorphological studies. GT design of radiological ILLD severity evaluation scale and analyzed radiological data. ED, OS, AR, DY, AL, VB provided clinical care. AM performed statistical analyses.

## Conflict of Interest

The authors declare that the research was conducted in the absence of any commercial or financial relationships that could be construed as a potential conflict of interest.

The handling editor declared a past collaboration with one of the authors, AR.

## Publisher’s Note

All claims expressed in this article are solely those of the authors and do not necessarily represent those of their affiliated organizations, or those of the publisher, the editors and the reviewers. Any product that may be evaluated in this article, or claim that may be made by its manufacturer, is not guaranteed or endorsed by the publisher.
