# Supplementary material for: Rituximab and Abatacept Are Effective in Differential Treatment of Interstitial Lymphocytic Lung Disease in Children With Primary Immunodeficiencies
Source: Front Immunol. 2021 Sep 9;12:704261. doi: 10.3389/fimmu.2021.704261 (PMC8458825; doi:10.3389/fimmu.2021.704261)
Supplement: Supplementary file 1 [file DataSheet_1.pdf]

Patients	Age at the start of therapy, years	Age of ILLD manifestation, years	Gene/ mutation	Histological /CT form of ILLD	Preceding immuno-suppressive therapy	ILLD clinical symptoms, points		ILLD CT-symptoms, points		Effect of abatacept or rituximab ILLD treatment at 12 months	Long-term treatment (>12 months)	ILLD/patient status
						0 mo	12 mo	0 mo	12 mo			
**Rituximab**												
P1	16	10	unknown	NLH/ NLH	Steroids	0,5	0,5	37	13	Remission	Off preparation	Remission
P2	7	4	*CTLA4*, c.410C>T, het	FB+NLH/ FB+NLH	Steroids, MTX, Aza, sirolimus, adalimumab, budesonide	0,5	0,5	29	9	Partial remission	Switched to abatacept	Remission
P3	7	3,5	22q del	No biopsy/ FB+NLH	Steroids, MMF	2,2	0,5	14	4	Remission	Off preparation	Remission
P4	16	12	*NFkB2*, c.39+1 G>A, het	FB+NLH/ FB+NLH	Steroids, MMF	2,2	0,5	32	14	Partial remission	Switched to abatacept	Remission
P5	8	6	*NBS*, c.657_661del, hom	NLH/ FB+NLH	No	4,2	0,5	36	12	Remission	Off preparation post HSCT	Died from HSCT complications
P6	18	17	*DCLRE1C*, c.1478_1479delTT; Del chr10	FB+LIP/ FB+LIP	Budesonide	1,0	0,5	24	20	No effect	Off preparation prior to HSCT	Died from HSCT complications

P7	3	2,5	*LRBA*, c.1359G>C, hom	No biopsy/ FB+NLH	Steroids, MMF	2,7	0,5	24	12	Partial remission	Switched to abatacept	Remission
P8	15	12	22q del	NLH/ FB+NLH	No	1,0	0,5	30	12	Remission	Off preparation	Remission
P9	15	14	22q del	No biopsy/ FB+NLH	Steroids, Aza	2,2	0,5	14	3	Remission	Lost to follow-up	Lost to follow-up
P10	14	13	unknown	FB+NLH/ FB+NLH	No	1,0	0,5	34	14	Remission	Off preparation	Remission
P11	11	7	*NFkB1*, c.1750-10C>G, het	FB+NLH/ FB+NLH	Steroids, budesonide	5,0	1,0	24	10	Partial remission	Switched to abatacept	Remission
P12	16	14	unknown	No biopsy/ FB+NLH	No	6,0	0,5	34	20	Remission	Lost to follow-up	Lost to follow-up
P13	17	16	unknown	FB+NLH/ FB+NLH	No	2,2	0,5	24	6	Remission	Off preparation	Remission
P14	15	12	unknown	FB+NLH/ FB+NLH	Sirolimus	2,2	0,5	30	6	Remission	Off preparation	Remission
P15	12	7	unknown	FB+NLH/ FB+NLH	Steroids	2,2	0,5	24	6	Partial remission	On preparation	Remission
P16	10	7	unknown	FB+NLH/ FB+NLH	Budesonide	3,8	1	28	8	Partial remission	On preparation	Remission

**Abatacept**												
P17	10	8	*ATM*, c.3928C>T, hom	LIP/LIP	Rituximab (4 inf)	4,1	1	31	24	Remission	Lost to follow-up	Lost to follow-up
P18	7	4	*ATM*, c.7088delA, hom	LIP/LIP	Steroids	2,8	0,5	12	3	Remission	Off preparation	Remission
P19	11	10	*ATM*, c.756_757delGT;c.*2756T>C	LIP/ FB+LIP	Steroids	4,7	2,8	35	20	Remission	Lost to follow-up	Lost to follow-up
P2*	8	4	*CTLA4*, c.410C>T, het	No biopsy/ FB+ LIP	Steroids, MTX, Aza, sirolimus, adalimumab, budesonide, rituximab	1,5	0,5	12	6	Remission	Off preparation post HSCT	Remission, chronic GVHD
P20	6	6	*ATM*, c.4777-2A>C, hom	LIP/LIP	Steroids, MTX	4,0	0,5	19	6	Remission	Off preparation, relapse after 3 months. Back on Abatacept	Remission
P21	16	13	*LRBA*, c.1568dupA hom	FB+LIP/ FB+LIP	Steroids, Aza, MMF, sirolimus	3,3	0,5	19	9	Remission	On preparation	Remission

P22	15	15	*CTLA4*, c.567G>A het	No biopsy/ FB+LIP	MMF	1	0,5	16	6	Remission	Off preparation post HSCT	Died from HSCT complications
P4*	17	12	*NFkB2*, c.39+1 G>A, het	No biopsy/ FB+LIP	MMF, rituximab	1	0,5	16	4	Remission	On preparation	Remission
P23	12	9	unknown	LIP/LIP	Steroids, rituximab (4 inf)	4,6	1	34	12	Remission	Lost to follow-up	Lost to follow-up
P24	17	15	*CTLA4*, c.118G>A het	FB+LIP/ FB+LIP	Steroids	2,1	0,5	11	2	Remission	On preparation	Remission
P25	6	5	*ATM*, c.2554C>T; c.9088G>A	No biopsy /LIP	No	2,1	0,5	9	6	Remission	Off preparation, relapse after 3 month. Back on Abatacept	Remission
P26	10	10	*ATM*, c.5036G>A; del ex50+intr50	No biopsy /LIP	No	2,8	0,5	8	2	Remission	On preparation	Remission
P27	6	6	*ATM*, c.7159_7160insAGCC, hom	LIP/ FB+LIP	No	5,3	1	24	12	Remission	Off preparation, relapse. Back on Abatacept	Remission

P28	5	4	*CTLA4*, c.670T>C, het	FB+LIP/ FB+LIP	Budesonide	3,1	0,5	28	9	Remission	On preparation	Remission
P29	8	6	*ATM*, c.5932 G>T, c.7835_7836dupGG	FB+LIP/ LIP	Budesonide	3,1	0,5	19	6	Remission	On preparation	Remission
P7*	4	2,5	*LRBA*, c.1359G>C, hom	No biopsy/ FB+LIP	Steroids, MMF, rituximab	3,1	0,5	14	6	Remission	Off preparation post HSCT	Remission, full immune reconstitution
P11*	12	7	*NFkB1*, c.1750-10C>G, het	FB+LIP/ FB+LIP	Steroids, budesonide, rituximab	1,0	0,5	12	6	Remission	On preparation	Remission

Table E1. Characteristics of patients with ILLD treated with rituximab or abatacept.

Abbreviation:

PID- primary immunodeficiency, CID – combined immunodeficiency, ILLD – interstitial lung disease, DGS – DiGeorge syndrome, NBS – Nijmegen breakage syndrome, A-T – Ataxia-telangiectasia, FB – follicular bronchiolitis, NLH – nodular lymphoid hyperplasia, LIP – lymphoid interstitial pneumonia MTX – methotrexate, AZA – azathioprine, MMF- mycophenolate mofetil, HSCT – hematopoietic stem cell transplantation, GVHD – graft versus host disease, P - patients in Group 1 who were switched to abatacept,*

Symptom	Points

	6	4	2	0.5
Cough	severe	moderate	minimal	none
Dyspnea	RF Gr3<br><br>SaO_2 <75%	RF Gr2<br><br>SaO_2 89-76%	RF Gr1<br><br>SaO_2 90-98%	none<br><br>SaO_2 98-100%
Rails	severe	moderate	minimal	none

Table E2. ILLD clinical symptoms evaluation scale.

Abbreviation: *RF – respiratory failure, Gr1 – grade1, Gr2 – grade2, Gr3 – grade 3.*

**Symptom**	**Points**			
	0	1	2	3
Focal lesions	none	Single (<10)	Multiple (>10)	
		*single new lesions	*multiple new lesions	

Interstitial changes	Lobe intact	Lobe partly involved	Total lobe involvement	
Lobe pneumatization	Normal	Mild decrease (ground-*glass* opacity)	Moderate decrease	Severe decrease (consolidation)
Fibrosis	Lobe intact	Lobe partly involved	Total lobe involvement	
Mediastinal lymph nodes	Not increased	Marginal increase (8-9 mm in diameter)	Increased (≥10 mm in diameter)	
Hilar lymph nodes	Not increased	Marginal increase (8-9 mm in diameter)	Increased (≥10 mm in diameter)	

Table E3. Radiological ILLD severity evaluation scale.

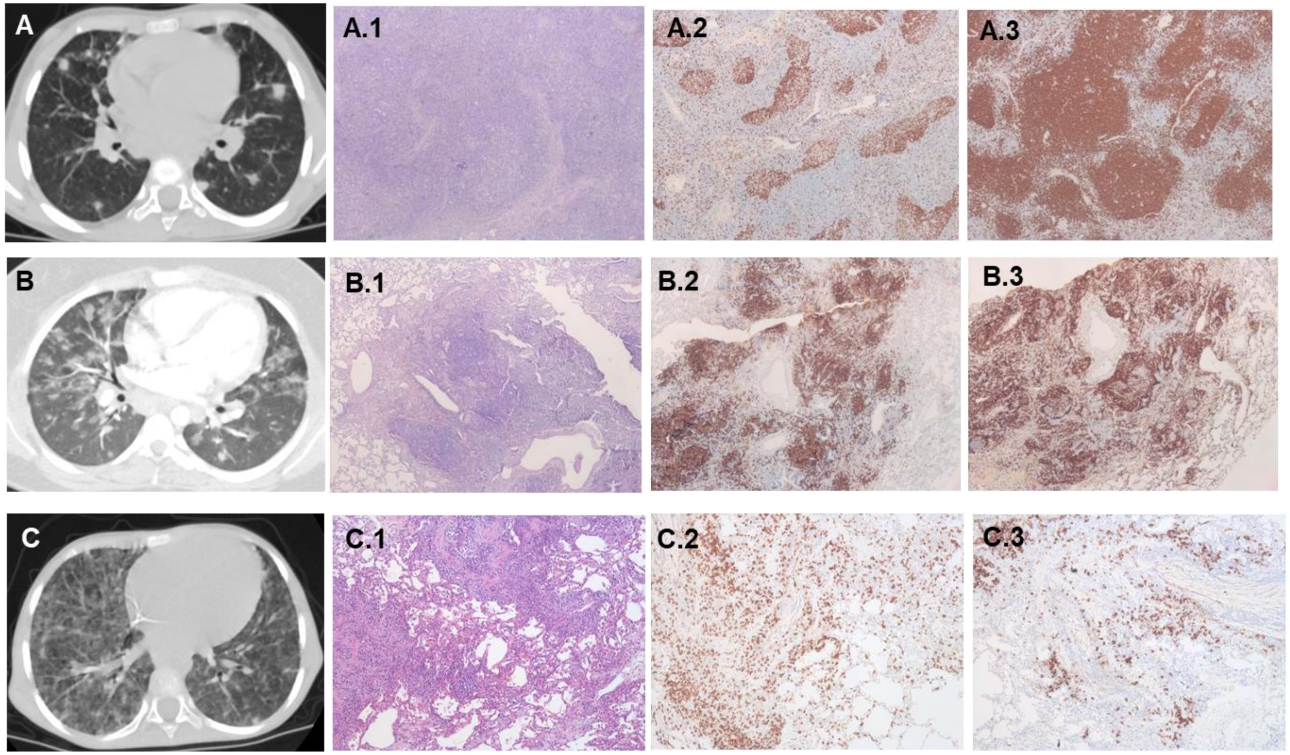

Figure E1. Radiological and histological picture of the pathomorphological forms of ILLD.

A, A.1 (H&Ex60), A.2 (CD3), A.3 (CD20) - sarcoid-like granulomas with predominant CD20 expression characteristic of NLH;

B, B.1 (H&Ex60), B.2 (CD3), B.3 (CD20) - diffuse peri-bronchial infiltration with CD3+ lymphocytes and lymphoid hyperplasia with germinal centers formation and high expression of CD20, characteristic for combination of FB and LIP;

C, C.1 (H&Ex200), C.2 (CD3), C.3 (CD20) - diffuse interstitial infiltration with CD3+ lymphocytes characteristic of LIP. H&E - hematoxylin and eosin, X – magnification.

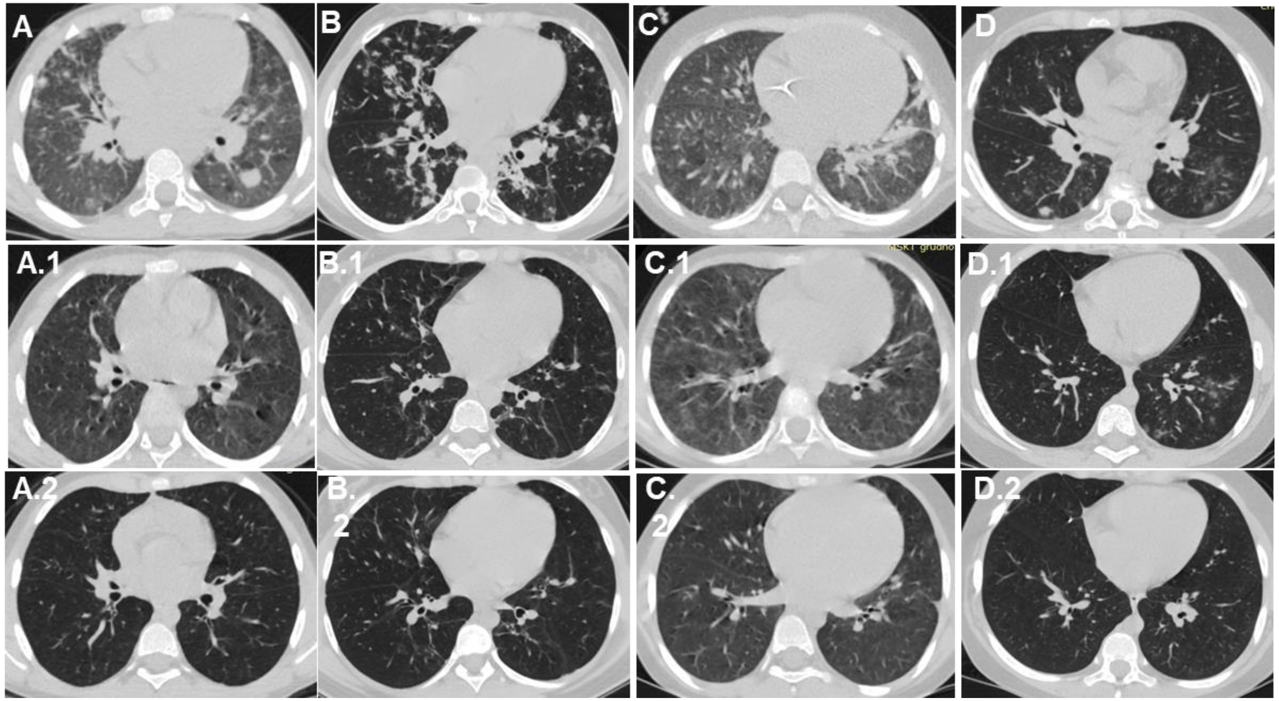

Figure E2. Dynamics of the CT-symptoms in ILLD patients switched to Abatacept.

A (P2) - before Rituximab, A.1 – before Abatacept, A.2 – on Abatacept; B (P4) - before Rituximab, B.1 – before Abatacept, B.2 – on Abatacept;

C (P7) - before Rituximab, C.1 – before Abatacept, C.2 – on Abatacept; D (P11) - before Rituximab, D.1 – before Abatacept, D.2 – on Abatacept.
